# The Association between Bile Salt Export Pump Single-Nucleotide Polymorphisms and Primary Biliary Cirrhosis Susceptibility and Ursodeoxycholic Acid Response

**DOI:** 10.1155/2014/350690

**Published:** 2014-10-19

**Authors:** Rui-rui Chen, Yuan-jun Li, Xin-min Zhou, Lu Wang, Juan Xing, Shuang Han, Li-na Cui, Lin-hua Zheng, Kai-chun Wu, Yong-quan Shi, Zhe-yi Han, Ying Han, Dai-ming Fan

**Affiliations:** ^1^Xijing Hospital of Digestive Diseases, State Key Laboratory of Cancer Biology, Fourth Military Medical University, Xi'an 710032, China; ^2^Department of Aerospace Medicine, Fourth Military Medical University, Xi'an 710032, China; ^3^College of Urban and Environmental Sciences, Peking University, Beijing 100871, China

## Abstract

*Background*. Primary biliary cirrhosis (PBC) is a chronic and progressive cholestasis liver disease. Bile salt export pump (BSEP) is the predominant bile salt efflux system of hepatocytes. BSEP gene has been attached great importance in the susceptibility of PBC and the response rate of ursodeoxycholic acid (UDCA) treatment of PBC patients. *Methods*. In this study, TaqMan assay was used to genotype four variants of BSEP, and the Barcelona criteria were used for evaluating the response rate of UDCA treatment. *Results*. Variant A allele of BSEP rs473351 (dominant model, OR = 2.063; 95% CI, 1.254–3.393; *P* = 0.004) was highly associated with PBC susceptibility. On the contrary, variant A allele of BSEP rs2287618 (dominant model, OR = 0.617; 95% CI, 0.411–0.928; *P* = 0.020) provided a protective role and Barcelona evaluation criterion indicated that the frequency of variant allele at BSEP rs2287618 was significantly decreased in UDCA-responsive PBC patients (*P* = 0.021). *Conclusion*. These results suggested that BSEP rs473351 was closely associated with the susceptibility of PBC and if people with BSEP rs2287618 were diagnosed as PBC, the UDCA treatment was not satisfactory. Larger studies with mixed ethnicity subjects and stratified by clinical and subclinical characteristics are needed to validate our findings.

## 1. Introduction

Primary biliary cirrhosis (PBC) is a kind of progressive chronic nonsuppurative cholangitis, mediated by disorder of the immune system [1, 2]. Pathological changes of PBC include biliary duct distraction, portal inflammation, and liver necrosis. It may cause intrahepatic bile duct injury and intrahepatic cholestasis, which finally leads to hepatic fibrosis and cirrhosis. The incidence of PBC increased in recent years due to lifestyle changes and cognitive rise of doctors. It is thought that the morbidity of PBC is affected by environmental factors and genetic factors [3, 4]. Many reports showed that there was an obvious familial aggregation of PBC. The prevalence of PBC clustering in a representative family with affected patients would reach a rate of 18.1, which is much higher than that in the general population [5]. In addition, the reported 63% concordance rate among monozygotic twins compared to the null concordance among dizygotic pairs [6], scoring as the highest among autoimmune diseases [5], indicated that gene susceptibility played an important role in the pathogenesis of PBC.

Gene susceptibility loci had been widely screened by researchers worldwide, and polymorphisms of different genes seemed to be associated with the development of PBC among different populations [7–10]. In the context of PBC, four genome-wide association studies (GWAS) have been performed and included homogenous groups from Northern American, Italian, mostly Northern American, and British patients, respectively. In addition to the HLA II genes, GWAS discovered 23 non-HLA susceptibility genes for PBC. Among these, IL12A, IL12RB2, IRF5, TNPO3, DENND1B, SPIB, TNFAIP2, CXCR5, CLEC16A, RAD51L1, IRF8, RPS6KA4, MAP3K7IP1, TNFRSF1A, PLCL2, ELMO1, and ARF7 played an important role in the morbidity of PBC in European ancestries, while TNFSF15 and POU2AF1 were unique in Japanese ancestries, and CD80, IKZK3, Il7R, NFKB1, and STAT4 were proved to be associated with PBC in both races. The disparity of genes among Europeans and Asians indicated that the results of Western countries could not apply to Chinese population in haste.

We carried out the overwhelming literature search to identify the possible genes that contributed to PBC susceptibility and analyzed single nucleotide polymorphisms (SNPs) for selecting genes which may participate in the pathogenesis of PBC for further research [11–13]. Finally, we found SNPs in bile salt export pump (BSEP) gene were highly associated with PBC susceptibility. BSEP, an ABC transmembrane transporter (ABCB 11), was the predominant bile salt efflux system of hepatocytes and mediates the cellular excretion of numerous conjugated bile salts [14].

In this study, we explored the association between four polymorphisms of BSEP (rs52304393, rs473351, rs860510, and rs2287618) and the susceptibility of PBC in Chinese population. Meanwhile, we analyzed the relationship between clinical indexes such as AMA, ALP, GGT, TBIL, and BSEP polymorphisms. Furthermore, the association between ursodeoxycholic acid (UDCA) response rate and BSEP polymorphisms was explored through logistic regression analyses.

## 2. Patients and Methods

### 2.1. Study Design

BSEP (ABCE 11) was located in chr2 and the four SNPs (rs52304393, rs473351, rs860510, and rs2287618), evaluated in this paper, were selected based on linkage disequilibrium and haplotype blocks from all SNPs (>5% minor allele frequency) of BSEP, containing 108.4 kb stream regions on the HapMap CHB data (http://hapmap.ncbi.nlm.nih.gov/index.html.zh), using the default setting of the Haploview 4.1 software program (Broad Institute, Cambridge, MA, USA).

### 2.2. Study Subjects

We analyzed a total of 448 subjects (134 PBC patients and 314 healthy controls) (Table 1). There were 114 women among 134 patients clinically diagnosed with PBC in Xijing Hospital, Xi'an, China, which is the largest comprehensive hospital in northwest of China, in the period 2004/07–2013/10. The 314 control subjects all were volunteers without any positive autoantibodies using indirect immunofluorescence test as well as abnormal biochemical indexes such as alanine aminotransferase (ALP) or *γ*-glutamyltranspeptidase (GGT). The age of patients ranged from 30 to 83 and that of the healthy subjects ranged from 12 to 71. The racial background of all subjects was Chinese. The diagnosis of PBC was based on criteria from the American Association for the Study of Liver Diseases [15]. Serum AMA, specific for the pyruvate dehydrogenase complex-E2 component, was measured by the enzyme-linked immune sorbent assay as reported previously [16]. UDCA response or no-response according to the criterion of Barcelona is defined as a decrease in ALP level >40% of the baseline level or a normal level and UDCA response means achieving the above standard after 1-year treatment [17]. An index of greater than seven was considered a positive result. All participants provided informed written consent for this study, which had been approved by the institutional ethics committee.

### 2.3. Genotyping

Genomic DNA from patients and controls was isolated using DNA extraction kit provided by SIGMA Tedia Company Inc. The four BSEP SNPs examined in this study (rs52304393, rs473351, rs860510, and rs2287618) were genotyped using TaqMan SNP Genotyping Assay according to the manufacturer's instructions, which were read with an ABI PRISM 7900HT sequence detection system (Applied Biosystems, Foster City, CA). Polymerase chain reaction was performed with a TaqMan Assay for real-time PCR (7500 real-time PCR system; Applied Biosystems) following the manufacturer's instructions.

### 2.4. Statistical Analysis

The Hardy-Weinberg equilibrium (HWE) test, which was a rule to check whether observed genotypic frequencies and allele frequencies between parents and their offspring were in a population, was used for each SNP, and any studies control group not conformed to the HWE test would be excluded. In other way, if the *P* value for HWE test <0.05, the study would be ruled out. The significance of allele distribution between PBC patients and healthy controls was assessed using the *χ* ^2^-test with the use 2 × 2 or 2 × 3 comparisons. Fisher's exact probability test was used for groups with fewer than 5 samples. The haplotype structure and gene interaction between the four examined BSEP SNPs (rs52304393, rs473351, rs860510, and rs2287618) was evaluated using multifactor dimensionality reduction (MDR), and we analyzed allele of 4 BSEP SNPs in different comparative models (dominant model, recessive model, and codominant model). A *P* value less than 0.05 was considered statistically significant. Logistic regression analysis [18] was used to assess the relationship between clinical indexes, UDCA response rate, and AMA and BSEP polymorphisms, respectively. All of the statistical analyses were performed using Stata version 12.0 (Stata Corporation, College Station, TX). Linkage disequilibrium (LD) for rs52304393, rs473351, rs860510, and rs2287618 was analyzed with the software Haploview 4.2.

## 3. Results

In both PBC patient and control group, the genotypes of selected polymorphisms of BSEP were in Hardy-Weinberg equilibrium, with no significant difference (Table 2).

**Figure 1:**
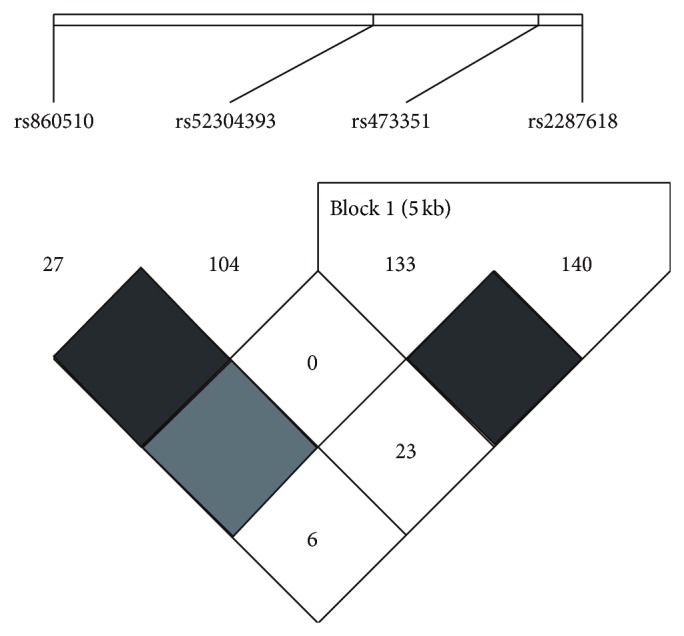
Linkage disequilibrium plot of BSEP SNPs in patients with PBC. *D*′ corresponding to each SNP pair are expressed as a percentage and shown within the respective square. Higher *D*′ is indicated by a brighter dark color.

Based on the genotypic concordance, two SNPs in BSEP gene (rs473351 and rs2287618) were detected in linkage disequilibrium (*D*′ > 0.9) (Figure 1). Haplotype of GG was the risk allele, which indicated that individuals carrying GG haplotype may suffer from PBC easier (Table 3).

All the loci selected were analyzed in dominant, recessive, and codominant genetic models, respectively. The rs473351 locus of BSEP was associated with PBC susceptibility. Its variant of A allele (dominant model, OR = 2.063; 95% CI, 1.254–3.393; *P* = 0.004) was highly associated with PBC susceptibility. The rs2287618 locus provided a protective role for PBC. The frequency of the minor A allele at rs2287618 was significantly decreased (dominant model, OR = 0.617; 95% CI, 0.411–0.928; *P* = 0.020) in PBC patients compared with controls (Table 4).

Ninety-six patients were included to analysis the relationship between clinical indexes such as AMA, ALP, GGT, and TBIL; UDCA response rate; and BSEP SNPs. Logistic regression analyses revealed that (1) the AMA-positive and AMA-negative population have a significant difference between the two groups with the rs2287618 mutation (*P* < 0.05). (2) The frequency of variant allele at rs2287618 was significantly associated with a high serum ALP (*P* = 0.04). (3) Two different evaluation criteria of UDCA response rate showed no significant difference, and the evaluation criterion of Barcelona indicated that the frequency of variant allele at rs2287618 was significantly decreased in UDCA-responsive PBC patients compared with UDCA-nonresponsive PBC patients (38.6% versus 53.4%; *P* = 0.021; OR = 0.55; 95% CI, 0.33–0.91) (Table 5).

## 4. Discussion

It was the first time to investigate the relationship between BSEP gene polymorphisms and PBC susceptibility in Chinese population. A variety of statistical models and genetic models have demonstrated that the BSEP mutations may play an important role in the process of PBC. This present study revealed that positivity for the rs473351 locus of BSEP was significantly higher in patients with PBC than in healthy subjects. SNPs of rs473351 and rs2287618 in BSEP gene are linked closely. Haplotype of GG was the risk allele, which indicated that individuals carrying GG haplotype may suffer from PBC easier.

The result showed that BSEP played an important role in the biliary excretion, and it was firstly found that gene polymorphisms of rs2287618 have an important role on the expression of the AMA. It was showed a significantly higher serum ALP, GGT, and bilirubin levels in the early stage of PBC. ALP was a kind of on-specificity alkaline phosphatase, which would rise in serum if damage happened to liver. Serum ALP appears as a useful prognostic marker for prediction of adverse outcome in the early stage PBC and deserves particular attention as a key surrogate inclusion marker for future therapeutic trials [19]. GGT was an enzyme produced mostly by live, and PBC patient's serum GGT was higher due to intrahepatic cholestasis. ALP, GGT, and bilirubin act as reference frame for PBC diagnosis, and the sensitiveness was 74.2%, 62.4%, and 82.2%, respectively [20]. ALP-regression analysis showed that PBC patients carrying rs2287618 risk allele have a significantly higher level of ALP than the PBC patients carrying other alleles of the same genes.

Genotype-guided dosing had been used for individual therapy in the recent years, and genotype-guided dosing of Warfarin, for example, showed a good result [21]. UDCA was regarded as the standard treatment of PBC [15, 22, 23]. The long-term prognosis for PBC patients is not different from an age- and sex-matched normal population when they adequately respond to UDCA after 1-year treatment as evaluated by Barcelona, Paris, and Toronto criteria. Response to UDCA is observed in about 60–65% of patients with PBC [17, 24, 25]. The response rate of UDCA should relate to one or more genes, which have not excogitated yet. In present research, patients carrying rs2287618 allele were significantly lower than those carrying protective alleles in response to UDCA, and our genetic study aim at the response rate of UDCA may provide reference for selecting genes and SNPs related to response rate of UDCA in the future.

The critical importance of BSEP to normal human hepatic function was illustrated by the progressive familial intrahepatic cholestasis (PFIC), a group of rare inherited disorders characterized by progressive liver disease that typically appear in childhood. Loss of function mutations in ABCB11 has been identified in patients with PFIC type 2 (PFIC2), typically resulting in absence of hepatic BSEP expression [26, 27]. These infants typically present with severe jaundice, hepatomegaly, failure to thrive, and pruritus [28, 29]. There was a rapid progressive course to cirrhosis and liver failure, which can only be cured by liver transplantation. The study of Takeyama et al. [30] pointed out that the mRNA expression levels of sodium taurocholate cotransporting polypeptide (NTCP), BSEP, and hepatic cholesterol 7*α*-hydroxylase (CYP7A1) were significantly higher in the PBC patients than in the controls (*P* < 0.01). The mRNA levels of NTCP and BSEP were significantly higher in the end-stage PBC patients than in the controls (*P* < 0.01). However, a study on the Caucasian population did not support the strong role of BSEP and MDR3 genetic variations in the pathogenesis of PBC and primary sclerosing cholangitis (PSC) [31]. Zollner et al. [32] found that, compared to controls, basolateral uptake systems (NTCP, OATP2) were reduced and BSEP and MRP2 were preserved, while MDR1, MDR3, and the basolateral efflux pump MRP3 were increased in PBC. Recent studies suggested that additional mutations in ABCB11 may be associated with various disease processes such as benign recurrent intrahepatic cholestasis (BRIC), intrahepatic cholestasis of pregnancy (ICP), and risk for drug-induced cholestasis (DC) [33–36].

In conclusion, we found that BSEP gene polymorphisms had a modest but significant association with susceptibility to PBC in the Chinese population. People with BSEP rs2287618 seemed not to suffer from PBC, and if people with BSEP rs2287618 were diagnosed as PBC, the UDCA treatment was not satisfactory. Connection between genetic variants and the function of the BSEP gene remains to be addressed in future investigations.

## Figures and Tables

**Table 1:** Demographic and clinical data of patients with PBC and controls.

Characteristics	PBC (134)	Control (314)
Age	58 (30–83)	40 (12–71)
Female/male	114/20	242/72
AMA positive, *n* (%)	128 (95.5%)	NA
UDCA treatment, *n* (%)	134 (100%)	NA
Liver transplantation, *n* (%)	0 (0%)	NA

PBC: primary biliary cirrhosis; AMA: antimitochondrial antibody; UDCA: ursodeoxycholic acid; NA: not available.

**Table 2:** Distribution of BSEP genotypes and alleles among PBC patients and controls.

SNP	Allele		Case					Control					HWE	
	A	B	AA	AB	BB	A	B	AA	AB	BB	A	B	*χ* ^2^	*P*
rs52304393	C	T	5	40	89	50	218	14	110	190	138	490	0.146	0.702
rs473351	A	G	1	36	97	38	230	2	44	268	48	580	0.018	0.895
rs860510	T	G	17	66	51	100	168	32	149	133	213	415	1.077	0.299
rs2287618	A	G	7	49	78	63	205	18	108	188	144	484	0.227	0.634

HWE: Hardy-Weinberg equilibrium.

**Table 3:** BSEP SNPs haplotypes and PBC susceptibility.

Haplotype (Block1)	PBC Fre	Control Fre	OR (95% CI)	*P* value
AA	0.585938	0.729748	0.524 (0.394–0.727)	0.022
AG	0.067079	0.080679	0.779 (0.457–1.329)	0.359
GA	0.086476	0.078309	1.099 (0.664–1.818)	0.713
GG	0.260507	0.111264	2.743 (1.915–4.930)	0.003

Fre: frequency of haplotype.

**Table 4:** Summary of different comparative results.

SNP	Allele		Model	OR (95% CI)	*P* value
	A∗	B∗			
rs52304393	C	T	Dominant model (AA vs. BB + AB)	0.803 (0.528, 1.221)	0.304
			Recessive model (BB vs. AB + AA)	0.789 (0.509, 1.222)	0.287
			Codominant model (AB vs. AA + BB)	0.482 (0.589, 1.204)	0.345

rs473351	A	G	Dominant model (AA vs. BB + AB)	2.063 (1.254, 3.393)	**0.004**
			Recessive model (BB vs. AB + AA)	2.095 (1.445, 3.560)	**0.005**
			Codominant model (AB vs. AA + BB)	1.869 (1.164, 3.002)	**0.010**

rs860510	T	G	Dominant model (AA vs. BB + AB)	1.231 (0.813, 1.862)	0.326
			Recessive model (BB vs. AB + AA)	1.143 (0.787, 1.876)	0.376
			Codominant model (AB vs. AA + BB)	1.178 (0.86, 1.600)	0.296

rs2287618	A	G	Dominant model (AA vs. BB + AB)	0.617 (0.411, 0.928)	**0.020**
			Recessive model (BB vs. AB + AA)	0.764 (0.391, 0.936)	**0.010**
			Codominant model (AB vs. AA + BB)	0.802 (0.589, 1.092)	0.161

B∗: main allele; A∗: minor allele.

**Table 5:** The association between BSEP SNPs and UDCA response.

SNP	UDCA CR *n* = 64 (%)	UDCA NR *n* = 32 (%)	Adjusted∗ OR (95% CI)	*P* value
rs52304393				
CC + CT	28 (0.44)	13 (0.41)	1.34 (0.21–0.99)	0.58
TT	36 (0.56)	19 (0.59)	1.00	
rs473351				
AA + AG	14 (0.22)	6 (0.20)	1.63 (1.01–4.87)	0.90
GG	50 (0.78)	26 (0.80)	1.00	
rs860510				
TT + TG	40 (0.62)	18 (0.57)	1.32 (1.03–2.38)	0.22
GG	24 (0.38)	14 (0.43)	1.00	
rs2287618				
AA + AG	38 (0.60)	14 (0.43)	0.32 (0.25–0.65)	0.01
GG	26 (0.40)	18 (0.57)	1.00	

^*^By age and sex adjustment and analyzed by logistic regression analyses.
